# The association between childhood traumatic events and weight restoration in adult anorexia nervosa inpatient treatment: a longitudinal study

**DOI:** 10.1007/s40519-026-01851-7

**Published:** 2026-04-03

**Authors:** Leonard Meyer-Schwickerath, Anna Suling, Bernd Löwe, Sarah Kottich, Angelika Weigel

**Affiliations:** 1 Department of Psychosomatic Medicine and Psychotherapy, University Medical Center Hamburg–Eppendorf, Martinistraße 52, 20246 Hamburg, Germany; 2 Department of Medical Biometry and Epidemiology, University Medical Center Hamburg–Eppendorf, Hamburg, Germany; 3 Schoen Clinic Hamburg Eilbek, Hamburg, Germany

**Keywords:** Anorexia nervosa, Childhood traumatic events, Inpatient treatment, Weight restoration, Body mass index (BMI)

## Abstract

**Purpose:**

To examine the association between childhood traumatic events and weight restoration during specialized inpatient treatment for anorexia nervosa (AN).

**Methods:**

Within this naturalistic prospective exploratory single-center study, patients with AN were recruited between 2014 and 2019 from a specialized eating disorder inpatient unit. Childhood traumatic events were assessed using the Childhood Trauma Questionnaire Short Form (CTQ-SF) and the Adverse Childhood Experiences Questionnaire (ACE). Posttraumatic stress disorder (PTSD) was diagnosed via a clinical interview. A mixed linear model was used to analyze associations between childhood traumatic events and weight restoration, while adjusting for the confounders AN subtype, medication, psychological comorbidities, gender and age.

**Results:**

Sixty patients with AN (mean age 24.60 years, *SD* = 7.60; 95% female; 77% restricting subtype) participated. The mean body mass index (BMI) increase after 12 weeks was 2.48 kg/m^2^ (*SD* = 0.88). Six patients (10%) were diagnosed with PTSD. The mean CTQ-SF sum score was 43.78 (*SD* = 18.00), and 17 patients (28.33%) indicated a relevant exposure to adverse childhood experiences (ACE). There were no relevant associations between childhood traumatic events and weight restoration during inpatient treatment (CTQ-SF *b* = 0.00, 95% CI [−0.02, 0.02]; ACE *b* = −0.04, 95% CI [−0.16, 0.07]).

**Conclusion:**

In our sample childhood traumatic events were not associated with weight restoration during inpatient treatment for AN. Given the low prevalence of childhood traumatic events in the present sample, further studies are recommended to explore the potential impact of childhood traumatic events on weight restoration in AN to optimize clinical outcomes.

**Level of evidence:**

Level III: evidence obtained from well-designed cohort or case–control analytic studies.

## Introduction

Anorexia nervosa (AN) is a potentially life-threatening mental disorder with a profound impact on somatic health, social participation, and quality of life in affected individuals [1–3]. AN is characterized by self-induced weight loss, with or without binge–purge behaviors, driven by an intense fear of gaining weight and a disturbed body image [4]. The incidence of AN remained constant over the past decades [5] with a lifetime prevalence ranging from 0.1% to 3.6% among females and up to 0.3% among males [5, 6]. AN also entails considerable burdens for individuals and healthcare systems [2, 7, 8]. The etiology of AN is complex and multifactorial, encompassing biological, psychological, and socio-cultural factors, including traumatic events [1, 3]. In detail, childhood traumatic events, such as sexual, physical and emotional abuse or neglect have been identified as a significant yet non-specific risk factor, particularly [9–11] in the development and severity of the binge–purge subtype of AN [12–15]. Additionally, the overall prevalence of traumatic events in AN is high, affecting up to 95.2% of AN patients under the age of 25 years in one sample [14, 16, 17]. However, there is evidence to suggest that only one-third of patients are diagnosed with a PTSD [10, 17]. This discrepancy may be attributed to an insufficient diagnosis and the recognition that not all childhood traumatic events necessarily lead to PTSD [14, 18, 19]. Notably, traumatic events frequently occur prior to the onset of AN [14, 20, 21]. The aforementioned findings prompt further investigation into whether patients diagnosed with AN and a history of traumatic events encounter more difficulties in achieving adequate weight restoration. However, the interplay between childhood traumatic events and AN remains complex. Childhood traumatic events may include both, complex and major traumatic events. Several studies highlighted the importance to consider complex traumatic events, referring to cumulative, multiple traumatic events such as chronic emotional abuse, neglect or physical abuse and mixed types. Thereby, patients with complex traumatic events may not necessarily meet criteria of PTSD [22, 23]. A prolonged exposure to childhood traumatic events appears to lead to a dose-dependent increase in AN symptoms, psychiatric comorbidity, increased emotional dysregulation and enduring stress-related biological changes, such as an altered hypothalamus–pituitary–adrenal axis [24–27]. A so-called “maltreated eco-phenotype” of eating disorders has been described, proposing a more heterogeneous perspective on childhood traumatic events, taking into account cumulative, complex traumatic events and henceforth having different effects on biological, psychological and treatment outcomes [13]. Major traumatic events refer to severe, distinct events like sexual abuse that often meet criteria of PTSD [10]. The International Classification of Diseases of the World Health Organization (ICD-11) introduced the diagnosis of a complex PTSD (CPTSD) combining PTSD and additional criteria, i.e., emotional dysregulation or difficulties in sustaining relationships [28]. Recent studies began to investigate the effects of PTSD and CPTSD on eating disorders pathology [29, 30]. All mentioned associations between childhood traumatic events and more severe eating disorders pathology, emotional dysregulation, and psychiatric comorbidity may complicate treatment progress and potentially hinder weight restoration. Effective treatment for AN focuses on long-term weight restoration and normalization of eating behaviors, often requiring inpatient treatment [31–34]. However, inadequate weight restoration during inpatient care remains a common challenge, particularly in patients with the binge–purge subtype of AN (18, 26) and is associated with unfavorable long-term outcomes [35, 36]. Identifying factors that contribute to inadequate weight restoration is therefore crucial for improving treatment outcomes in AN. A recent study by Sjögren et al. has revealed no direct impact of lifetime traumatic events on early weight restoration in AN patients [37]. In the study, no influence of traumatic experiences on early weight gain during the first eight weeks of inpatient treatment for patients with AN was observed. However, a correlation was identified between traumatic experiences and more severe eating disorder pathology upon admission. It is important to note that lifetime traumatic experiences were assessed, rather than focusing specifically on childhood traumatic events. Studies investigating the influence of childhood traumatic events on weight restoration during adult AN inpatient treatment remain scarce [37]. To the best of our knowledge, no studies have focused exclusively on childhood traumatic events while concomitantly disentangling the complex interplay between eating disorder pathology, childhood traumatic events, and weight restoration during adult inpatient treatment. An in-depth understanding of this interplay could contribute to more effective and tailored inpatient treatment concepts [25]. Based on previous findings linking childhood traumatic events to greater AN severity, the present study explored whether childhood traumatic events are associated with weight restoration during adult inpatient treatment.

## Methods

### Study design and participants

This naturalistic prospective exploratory single-center study was conducted in a specialized eating disorder inpatient unit at Schoen Clinic Hamburg Eilbek, Hamburg, Germany. The inpatient treatment concept was based on cognitive-behavioral therapy (CBT) implemented by a multidisciplinary team including physicians, psychologists, nursing staff, nutritionists, art therapists and physiotherapists. Treatment modules comprised weight gain contracts (600–1200 g per week), nutrition counseling, food diaries, individual and group therapy sessions, psychoeducation on AN, supervised meals, training in social and emotional competence, bodily tension regulation, art therapy and physiotherapy. The treatment complied with German guidelines for eating disorders [38]. A trauma-specific treatment was not implemented.

### Participants

Eligible participants were aged ≥ 18 years, diagnosed with AN based on DSM-IV criteria requiring inpatient treatment. Proficiency in German was necessary to complete the questionnaires. Exclusion criteria for study participation were acute suicidality, substance abuse within the past three months, psychotic or bipolar disorder, life-threatening somatic conditions requiring acute specialized medical care. The study was approved by the competent Ethics Committee (Hamburg Medical Council PV4622, 28.07.2015).

### Recruitment

Participants were recruited between November 2014 and December 2019 upon admission to the inpatient unit. Eligible patients were informed verbally and in writing by a physician and signed an informed consent. Study participation had no impact on the treatment and participants did not receive any incentives.

### Measures

AN diagnoses and subtypes were confirmed using a Structured Clinical Interview based on the criteria of DSM-IV Axis I disorders [39]. As the German version of the DSM-5 was unavailable at the start of the study, the DSM-IV criteria were used throughout the entire study period to ensure diagnostic consistency. Comorbidities, including affective disorders, PTSD and personality disorders were assessed via clinical interviews upon admission. Additionally, medication and sociodemographic data including age, gender, family status, previous outpatient and inpatient treatments were assessed.

To evaluate childhood traumatic events, the German version of the Childhood Trauma Questionnaire Short Form (CTQ-SF) and the Adverse Childhood Experiences (ACE) were assessed at admission, as well as the PDS-d-1 (Posttraumatic Diagnostic Scale for DSM-IV) to obtain PTSD symptoms within the past month related to a traumatic event [40–47]. The PDS-d-1 was used to assess PTSD symptom severity and did not replace a clinical diagnosis. The German version of the Eating Disorder Inventory (EDI-2) was assessed at the time of admission and upon discharge to evaluate eating disorder pathology [48].

The German version of the CTQ-SF comprises 25 Likert-scale items (range: 1 = “Not True” to 5 = “Very Often True”), covering five subscales: Emotional abuse, Physical abuse, Sexual abuse, Emotional neglect and Physical neglect during childhood. Each subscale consists of five items (range 5–25 points) and a higher score indicates a greater severity of childhood trauma. An additional Minimization/Denial subscale consisting of three items identifies underreporting.

The German version of the ACE Questionnaire comprises ten items referring to adverse childhood events before the age of 18, each item with a dichotomous Yes/No answer: (1). Emotional abuse, (2). Physical abuse, (3). Sexual abuse, (4). Emotional neglect, (5). Physical neglect, (6). Separation from a parent, (7). Violence against mother, (8). Substance abuse of a household member, (9). Mental illness of a household member, (10). Imprisonment of a household member. Four or more “Yes” answers reflect a relevant exposure to adverse childhood events [49–51].

In this study, two retrospective, self-reported questionnaires were used to assess childhood traumatic events. The ACE measures a broad spectrum of childhood traumatic events, including separation from a parent and mental illness of a household member, which are not included in the CTQ-SF [45]. In contrast, the CTQ-SF assesses the severity of specific types of childhood traumatic events using multiple items for each type and Likert-scale ratings. In addition, the CTQ-SF identifies potential underreporting compared to the ACE. Using both questionnaires allows the assessment of a broad spectrum (ACE) and the severity (CTQ-SF) of childhood traumatic events while also reducing the likelihood that relevant traumatic events remain unassessed.

The PDS-d-1 (Sect. 3) questionnaire comprises 17 items and evaluates three symptom clusters: (1). Intrusions, (2). Avoidance, (3). Vegetative hyperarousal. Each item is rated on a scale of 0–3, ranging from 0 (“never or once in the past month”) to 3 (“5 times or more per week/almost always”). The maximum sum score is 51 points. The PDS-d-1 was included in this study to evaluate current PTSD-related symptoms, as PTSD is often underdiagnosed in clinical practice [10], yet the symptoms and their severity are clinically relevant.

The German version of the EDI-2 is a 91-item self-report questionnaire with Likert-scale responses (range: 1 = “Never” to 6 = “Always”), covering 11 subscales: (1). Drive for thinness, (2). Bulimia, (3). Body dissatisfaction, (4). Ineffectiveness, (5). Perfectionism, (6). Distrust, (7). Interoceptive perception, (8). Anxiety about maturation, (9). Asceticism, (10). Impulse regulation, (11). Social insecurity.

**Fig. 1:**
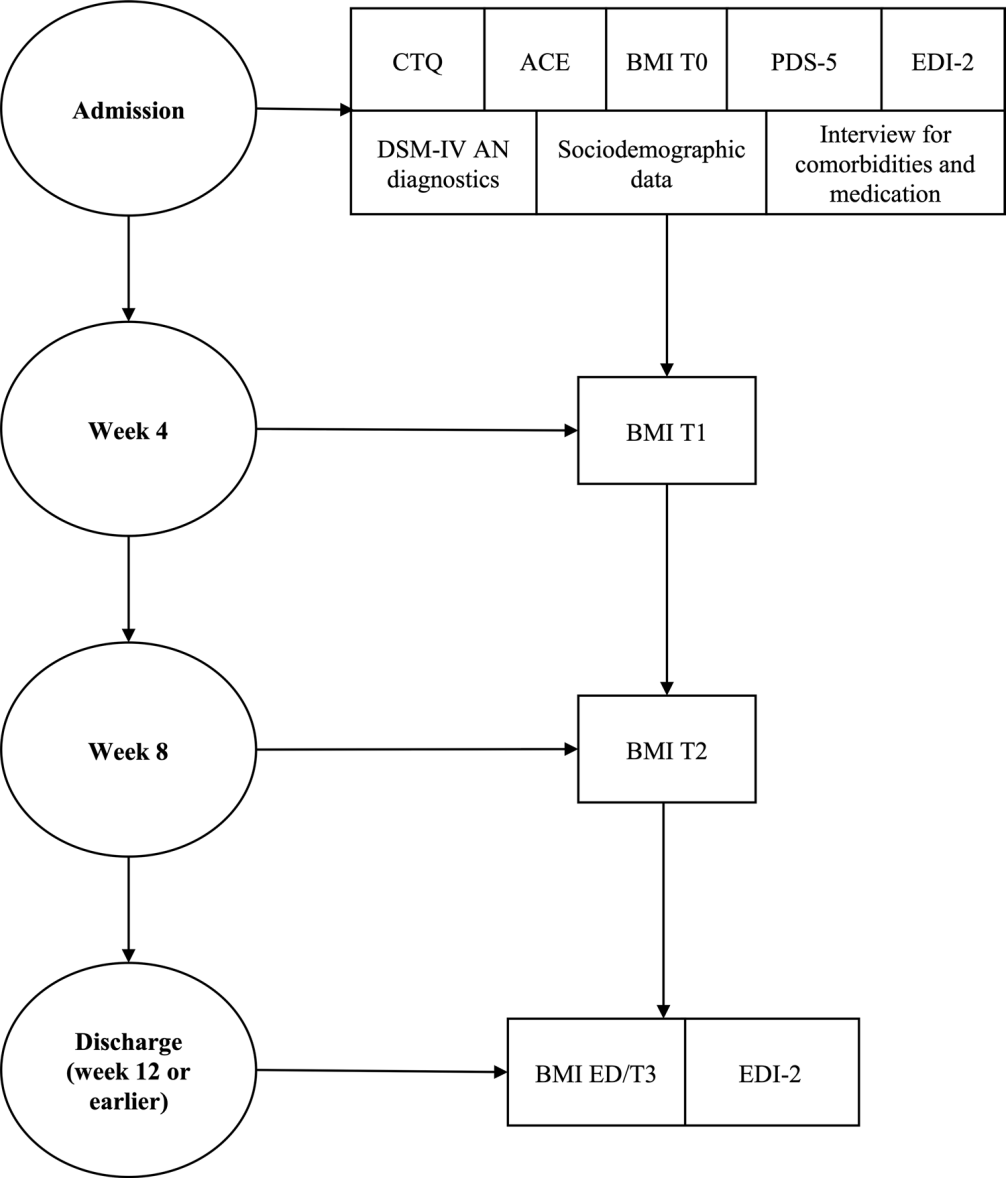
Study design including measures

Objective measurements of weight restoration were conducted by the inpatient staff on the day of admission and repeated regularly over the course of the inpatient treatment until discharge, depending on the treatment and weight gain plan. The body mass index (BMI) (kg/m^2^) was calculated based on weight (kg) and height (cm), the latter was measured only once at admission. For this study, weight was measured at admission (T0), week 4 (T1), week 8 (T2), week 12 (T3). In case of an inpatient treatment over 12 weeks, BMI measurements at week 12 were used. For participants discharged earlier than week 12 (ED), the last available weight measurement prior to discharge was used. Figure 1 visualizes the study design.

### Statistical analysis

Baseline data were analyzed descriptively for the total sample and separately for each AN subtype. Nominal variables were analyzed using number and percentage, metric variables were analyzed using mean and standard deviation (*SD*). The CTQ-SF, ACE and PDS-d-1 were examined for potential intercorrelation. A mixed linear model was used to examine a potential association between childhood traumatic events and weight restoration during inpatient treatment for AN. The independent variable of interest was CTQ-SF, while the relevant confounders, namely ACE, age, BMI at admission, AN subtype, gender, psychological comorbidity and medication, were included as further independent variables in the model. The dependent variable was defined as weight restoration over time, measured by changes in BMI from baseline (T0) to subsequent measurements (T1, T2, ED/T3). Two patients were discharged later than week 12 (ED/T3) and for these participants the week 12 measurement was used as it represents a reasonable and comparable timepoint within this sample. The analysis was furthermore controlled for AN subtype, gender, psychological comorbidity and medication. The patient was included as a random term to adjust for the dependent structure induced by multiple measurements per patient. Missing data in the outcome variables were not imputed but handled indirectly under the missing-at-random assumption via full-information maximum likelihood estimation. Residuals were visually inspected for normal distribution. For this study the mean sum scores of the CTQ-SF and ACE were used as a continuous variable for the mixed linear model.

An additional mixed linear model analyzed the association between childhood traumatic events and eating disorder pathology over the course of the inpatient treatment. The independent variable of interest was CTQ-SF, while the relevant confounders, namely ACE, age, EDI-2 at admission, AN subtype, gender, psychological comorbidity and medication, were included as further independent variables into the model. The dependent variable was defined as changes over time in eating pathology, measured by the difference between total EDI-2 sum scores at admission and discharge. The patient was included as a random term. In line with the exploratory design, no correction for multiple comparisons was applied and no fixed significance level was defined. Results are presented using effect estimates and 95% confidence intervals (CI). P-values are reported descriptively and are not to be interpreted in a confirmatory manner. Statistical analyses were performed using IBM SPSS Statistics (version: 28.0.1.0) [52].

## Results

### Sample characteristics

A total of 60 adult patients with AN were recruited between November 2014 and December 2019, of whom 77% (*n* = 46) had the restricting AN subtype. All 60 patients were included in the statistical analyses. The sample included 57 female (95%) and three male patients. The mean age of the total sample was 24.60 years (*SD* = 7.60) with no missing data. The mean BMI at admission was 15.58 kg/m^2^ (*SD* = 1.50), which was lower in patients with AN of the restricting subtype (*M* = 15.35 kg/m^2^, *SD* = 1.57) as compared to those with the binge–purge subtype (*M* = 16.31 kg/m^2^, *SD* = 1.01). BMI data were available for all participants at week 4, yet data availability decreased during the course of the study (loss to follow-up at week 8 18% (*n* = 11), 57% earlier than week 12 or at week 12 (n = 34), respectively). Six patients (10%) were clinically diagnosed with a PTSD with no missing data in the sample. The mean CTQ-SF sum score in this sample at admission was 43.78 (*SD* = 18.00, range 27–102 points) with higher scores in the binge–purge subgroup (*M* = 56.93, *SD* = 22.51) as compared to the restricting subgroup (*M* = 39.78, *SD* = 14.44). The mean ACE sum score at admission was higher in the group with the binge–purge subtype (restricting: *M* = 1.83, *SD* = 2.23; binge–purge: *M* = 4.14, *SD* = 3.01). There were no missing data for the CTQ-SF and the ACE in this sample. The mean PDS-d-1 sum score in this sample at admission was 21.90 (*SD* = 12.75), with a range from 0 to 43 points. There were 14 missing data in the restricting subtype and four missing data in the binge–purge subtype. Most participants (*n* = 42, 70%) were undergoing their first inpatient treatment and nearly half (*n* = 29, 48%) reported no previous outpatient treatment. Data for all participants were available regarding previous inpatient and outpatient treatment. The majority of patients (67%) were single, with data available for the total sample. During inpatient treatment, nine patients (15%) did not receive additional medication, 25 patients (42%) received only somatic medication, three patients (5%) were treated solely with psychotropic drugs and 23 patients (38%) received both drug groups. Data for the total sample were available regarding medication. Both AN subgroups were comparable in terms of age, PDS-d-1 sum score, PTSD prevalence, history of in- and outpatient treatment, gender, family status and medication (see Table 1 for sociodemographic characteristics).

**Table 1:** Demographic and clinical characteristics for each AN subtype

	Restricting (*n* = 46)	Binge–purge (*n* = 14)
Age *M* (*SD*)	23.96 (7.82)	26.71 (6.66)
BMI T0 (admission) *M* (*SD*)	15.35 (1.57)	16.31 (1.01)
CTQ-SF sum score *M* (*SD*)	39.78 (14.44)	56.93 (22.51)
ACE sum score *M* (*SD*)	1.83 (2.23)	4.14 (3.01)
PDS-d-1 sum score *M* (*SD*)^c^	20.06 (12.14)	27.80 (13.47)
PTSD N (%)	4 (8.70)	2 (14.29)
Previous inpatient treatment N (% Yes)	12 (26.08)	6 (42.85)
Previous outpatient treatment N (% Yes)	27 (58.70)	4 (28.57)
Gender N (% female)	43 (93.48)	14 (100.00)
Family status N (%)		
Single	31 (67.39)	9 (64.29)
Partnership	12 (26.09)	3 (21.43)
Married	3 (6.52)	2 (14.29)
Medication N (%)		
No medication	7 (15.22)	2 (14.29)
Somatic medication	23 (50.00)	2 (14.29)
Psychotropic drugs	2 (4.35)	1 (7.14)
Both drug groups	14 (30.43)	9 (64.29)

^c^ Variable containing missing values

AN, anorexia nervosa; BMI, body mass index; PTSD, posttraumatic stress disorder; CTQ-SF, Childhood Trauma Questionnaire Short Form; ACE, Adverse Childhood Experiences Questionnaire; PDS-d-1, Posttraumatic Diagnostic Scale for DSM-IV

### Association between childhood traumatic events and weight restoration

The mean body mass index (BMI) increase after 12 weeks was 2.48 kg/m^2^ (*SD* = 0.88). Weight restoration for each AN subtype during inpatient treatment is displayed in Table 2. The intercorrelation between the ACE and the CTQ-SF was high (*r* = 0.85). The mixed linear model revealed an effect of time on weight restoration in the total sample. The adjusted estimated marginal mean BMI increased by 0.88 kg/m^2^ (95% CI [0.46, 1.31]) at week 4, by 1.68 kg/m^2^ (95% CI [1.25, 2.11]) at week 8 and by 2.22 kg/m^2^ (95% CI [1.78, 2.66]) at discharge between weeks 8 and 12. Weight restoration did not differ between the restricting and binge–purge AN subtypes (*b* = 0.34, 95% CI [-0.04, 0.72]). Furthermore, no association between the CTQ-SF (*b* = 0.00, 95% CI [−0.02, 0.02]) or the ACE (*b* = −0.04, 95% CI [−0.16, 0.07]) and weight restoration was observed, indicating that childhood traumatic events were not associated with weight restoration in the investigated sample. None of the other included variables showed clinically relevant effects in the analysis (see Table 3).

**Table 2:** Weight restoration for each AN subtype

Change in BMI from baseline (T0)	Restricting (*n* = 46)	Binge–purge (*n* = 14)
∆BMI T1 *M* (*SD*), *n*	1.04 (0.52), 46	0.70 (0.38), 14
∆BMI T2 *M* (*SD*), *n*	1.93 (0.61), 36	1.38 (0.44), 13
∆BMI ED/T3 *M* (*SD*)^a^, *n*	2.60 (0.86), 23	1.59 (0.60), 3

^a^ Data including two participants who were discharged later than week 12 (ED/T3) and for these participants the week 12 measurement was used

AN, anorexia nervosa; BMI, body mass index (kg/m^2^); BMI T0, admission; BMI T1, week 4; BMI T2, week 8; BMI ED/T3; earlier discharge than week 12 or at week 12

**Table 3:** Estimates of fixed effects^a^ from the mixed linear model analyzing the association between childhood traumatic events and weight restoration

	Estimate (*b*)	Confidence interval 95%	*p*-value
Age	0.01	[−0.01, 0.03]	.588
BMI T0 (admission)	−0.14	[−0.24, -0.04]	.007
CTQ-SF sum score	0.00	[−0.02, 0.02]	.904
ACE sum score	−0.04	[−0.16, 0.07]	.424
AN subtype			
Restricting	0.34	[−0.04, 0.72]	.082
Binge–purge	Ref	Ref	Ref
Gender			
Female vs. male	−0.13	[−0.83, 0.57]	.705
Psychological comorbidity			
No vs. yes	−0.01	[−0.50, 0.49]	.984
Medication			
No medication	−0.03	[−0.48, 0.42]	.894
Somatic medication	−0.24	[−0.59, 0.11]	.172
Psychotropic drugs	−0.64	[−1.30, 0.02]	.057
Both drug groups	Ref	Ref	Ref
Weight restoration			
BMI T1	−1.33	[−1.50, −1.17]	< .001
BMI T2	−0.54	[−0.71, −0.37]	< .001
BMI ED/T3^b^	Ref	Ref	Ref

^a^Dependent variable: weight restoration over time, measured by changes in BMI from baseline (T0) to subsequent measurements (T1, T2, ED/T3)

^b^ Data including two participants who were discharged later than week 12 (ED/T3) and for these participants the week 12 measurement was used. Ref = reference (*b* = 0)

AN, anorexia nervosa; BMI, body mass index; BMI T0, admission; BMI T1, week 4; BMI T2, week 8; BMI ED/T3; earlier discharge than week 12 or at week 12; CTQ-SF, Childhood Trauma Questionnaire Short Form; ACE, Adverse Childhood Experiences Questionnaire

A separate mixed linear model analyzing the association between childhood traumatic events and eating disorder pathology during inpatient treatment also revealed no relevant effects of the CTQ-SF (*b* = 0.11, 95% CI [−1.69, 1.91]) or the ACE (*b* = 4.72, 95% CI [−6.84, 16.27]), indicating that childhood traumatic events were not associated with changes in eating disorder pathology during inpatient treatment. None of the other included variables revealed relevant associations in this analysis.

## Discussion

The present study investigated whether childhood traumatic events are associated with weight restoration during inpatient treatment for AN. Our results showed a clinically relevant weight restoration over the course of inpatient treatment, which was comparable in the restricting and the binge–purge AN subtypes. In the present study, no association between childhood traumatic events and weight restoration or eating disorder pathology was identified. These results align with recent studies reporting no direct impact of lifetime traumatic events on early weight restoration in AN patients [37]. The descriptive data revealed a higher prevalence of childhood traumatic events in the binge–purge subtype compared to the restricting subtype, which is consistent with current research suggesting that the binge–purge subtype is more frequently associated with childhood traumatic events [10, 14, 20]. Regarding comorbid PTSD diagnoses and PTSD symptoms, no differences were found between AN subtypes. However, these findings should also be interpreted in light of recent conceptualizations distinguishing between major and complex, cumulative forms of traumatic events and their potential heterogeneous impact on AN [10, 29, 30]. Previous studies have suggested that complex traumatic events, such as chronic emotional abuse or neglect, may be particularly relevant for eating disorder pathology, even in the absence of a formal PTSD diagnosis [13, 22]. In this context, the relatively low prevalence of PTSD diagnoses in our sample does not necessarily imply a low burden of trauma-related distress. Rather, trauma-related influences on AN may be complex and heterogeneous and not fully captured by PTSD diagnoses. Recent studies have indicated an indirect effect of childhood traumatic events on AN symptoms and treatment outcomes, including weight restoration, via mediating factors such as emotional dysregulation [13, 25, 26, 53, 54]. Eating disorder pathology may function as a maladaptive coping mechanism that reduces for example hyperarousal and distressing thoughts or feelings associated with traumatic events [18, 55]. Consequently, relinquishing eating disorder symptoms during treatment could provoke trauma-related distress or PTSD-related symptoms, representing a considerable obstacle for AN patients [18, 56]. However, these potential mediating mechanisms were not assessed in this study, which limits the explanatory power of the findings. Accordingly, indirect pathways linking childhood traumatic events and AN outcomes could be investigated in greater detail in future studies with longer follow-up periods. Further research addressing this complex interplay is warranted, given that AN is associated with severe complications and outcomes [31]. In this context, several limitations of this study should be considered. There was a substantial proportion of missing data at later timepoints, particularly at discharge, where dropout exceeded 50%. Although the missing outcome data were handled using full-information maximum likelihood estimation under the MAR assumption, such a high level of attrition inevitably reduces statistical precision and limits the robustness of the findings. It is important to note that no statistical method can fully compensate for the loss of information associated with this degree of missingness. Therefore, the results, especially those pertaining to discharge, should be interpreted with appropriate caution. Another limitation of this study is that relevant childhood traumatic events were underrepresented in the restricting subgroup, as mean sum scores for ACE and CTQ-SF were relatively low. While the binge–purge subgroup had higher mean sum scores, the sample size was relatively small. This limits the interpretability of the subgroup findings and statistical power for subgroup analyses. In addition, the overall variability of childhood traumatic events within the sample was limited, particularly at the higher end within the restricting subgroup. Therefore, the findings from this sample should be interpreted with caution and cannot be generalized to populations with a higher level of exposure to childhood traumatic events. Furthermore, the present study focused on short-term weight restoration during inpatient treatment. However, AN is often a severe, chronic disorder with high relapse rates [31, 54, 57–59]. Consequently, the current findings cannot be generalized to long-term outcomes, as long-term follow-up data were not available in this sample. Future studies should include larger samples that better represent the binge–purge subtype, and examine longer follow-up periods across different treatment settings, including outpatient care. This would improve the understanding of the role of childhood traumatic events in the course of AN treatment. In addition, the number of PTSD diagnoses was rather low in the complete sample (10%) compared to other studies reporting a range of 9%–17.2% [10, 18, 19]. Additionally, missing data on PDS-d-1 sum scores limited the ability to assess PTSD prevalence and severity. Another notable limitation of this study is that it did not differentiate between specific types of childhood traumatic events, especially the distinction between major and complex, cumulative childhood traumatic events, although previous research suggests different phenotypes and outcomes, such as weight restoration [51]. The mean sum scores of the ACE and CTQ-SF were used for calculations in order to address the main research question, instead of CTQ-SF subscale calculations or mean sum scores of specific items of the ACE. However, future research might focus on specific types, the severity and duration of childhood traumatic events and their impact on AN. Furthermore, a more detailed assessment of psychological comorbidities such as borderline personality disorder [9] was not conducted. Also, other outcomes, including specific eating disorders or general psychopathology, were not systematically evaluated. Therefore, it was not possible to examine whether childhood traumatic events were associated with these outcomes. Finally, as the majority of participants were female, findings may not be applicable to male patients with AN. Despite these limitations, to the best of our knowledge no study has yet examined the association between childhood traumatic events and weight restoration during inpatient treatment for AN. As the onset of AN is often during adolescence and traumatic events frequently occur before [21], it seems to be essential to examine childhood traumatic events. Moreover, despite the high correlation between the ACE and the CTQ-SF, it is nevertheless essential to examine a broad spectrum of childhood traumatic events in order to assess the co-occurrence of multiple traumatic events [46, 60, 61]. A broad spectrum of childhood traumatic events was addressed using two widely validated, retrospective, self-reported questionnaires: the ACE and the CTQ-SF. In addition, the utilization of the PDS-d-1 to evaluate PTSD-related symptoms appears to be a promising approach.

In summary, this study did not identify an association between childhood traumatic events and weight restoration during inpatient treatment for AN. However, considering the low prevalence of childhood traumatic events and PTSD diagnoses and symptoms in this sample, future studies should aim for larger samples, include more patients reporting childhood traumatic events and PTSD symptoms, make a clear distinction between major and complex traumatic events, recruit more male patients, and especially monitor long-term treatment outcomes after inpatient treatment. Furthermore, future research could assess indirect mediators between childhood traumatic events and AN, such as emotional dysregulation.

## Strength and limitations

The key strength of this study is its longitudinal design within a trauma non-specific inpatient treatment setting for eating disorders to examine the association between childhood traumatic events and weight restoration in adult patients with AN. However, the findings are limited by a substantial proportion of missing data at later timepoints, a relatively low prevalence of childhood traumatic events and PTSD diagnoses, and limited statistical power for subgroup analyses. Therefore, the results of this study should be interpreted with caution, particularly regarding generalizability and long-term outcomes.

## What is already known on this subject?

Childhood traumatic events are common among patients with AN and are associated with a dose-dependent increase in AN symptoms, psychiatric comorbidity, emotional dysregulation, and stress-related biological changes. Preliminary research indicates a potential unfavorable impact on treatment outcomes, although evidence regarding short-term inpatient weight restoration remains limited.

## What this study adds?

The present study identified no association between childhood traumatic events and weight restoration during inpatient treatment for AN. These findings suggest that childhood traumatic events may not directly affect short-term weight restoration in AN, while highlighting the necessity of investigating indirect mediators and long-term outcomes. These research efforts appear to be essential for developing more tailored inpatient treatments, optimizing outcomes for AN patients, and reducing healthcare costs.

## Data Availability

The datasets generated and analyzed during the current study are available from the corresponding author on reasonable request.
